# Small dose monitor based on silicon‐carbide diodes for FLASH radiotherapy

**DOI:** 10.1002/mp.70354

**Published:** 2026-02-27

**Authors:** Ivan Lopez Paz, Celeste Fleta, Ángela Henao, Sophie Heinrich, Consuelo Guardiola

**Affiliations:** ^1^ Institute of Microelectronics of Barcelona IMB‐CNM (CSIC) Barcelona Spain; ^2^ Institut Curie, Inserm U1021‐CNRS UMR 3347, University Paris‐Saclay PSL Research University, Centre Universitaire Orsay France

**Keywords:** dosimeters, silicon carbide, ultra‐high dose rate

## Abstract

**Background:**

The FLASH biological effect in radiotherapy has been observed to appear at ultra‐high dose rates UHDR (>40 Gy/s), where the accurate dosimetry at such high rates is still a challenge.

**Purpose:**

A new 4 × 4 array of SiC‐based detectors (1 mm diameter, 2.2 mm pitch) is proposed for dosimetry in UHDR, as well as the feasibility of a position sensitive technology demonstrator covering 7 × 7 ${\mathrm{mm}}^{2}$ placed on a movable micro‐stage to cover larger surfaces.

**Methods:**

In the ElectronFlash LINAC at the Institute Curie, two silicon carbide prototypes (a 2.2 mm diameter single diode and a 4 × 4‐array of 1 mm diameter with a pitch of 2.2 mm), biased at 0 V, are exposed to a 0.5–5 μs pulsed electron beam of 7 MeV alongside a flashDiamond PTW as reference dosimeter to characterize their response, time structure and position response.

**Results:**

A linearity better than 3.5% is observed up to 10 Gy per pulse of the single diode device only limited by the reference dosimetry. The pulse structure measured is consistent with the reference beam current transformer installed in the LINAC, allowing for instantaneous pulse discrimination at UHDR and its verification in the measurement point. Moreover, results demonstrate the viability of using SiC arrays to quantify the dose per pulse in a 70 × 50 ${\mathrm{mm}}^{2}$ area with a granularity of 1 × 2.2 ${\mathrm{mm}}^{2}$, paving the way to larger arrays and thus toward potential 2D dose monitoring.

**Conclusions:**

The possibility of a position sensitive SiC dose monitor for UHDR is demonstrated, as the technology demonstrator has been proven to maintain good linearity up to at least 10 Gy per pulse, with a time resolution enough to observe microsecond pulses and position sensitive readout.

## 1 INTRODUCTION

A new radiotherapy (RT) modality, called FLASH RT,^1^ is being investigated to reduce healthy tissue toxicities. It consists of the delivery of pulses with the ultra high dose rate (UHDR) (>40 Gy/s), which reduces the normal tissue complication probability from the long term complications associated to conventional dose‐rate at equal dose (e.g., senescence, inflammation, loss of function, i.a.), while maintaining similar tumor control probability to that of the conventional RT (∼0.05 Gy/s).^2^ Hence, a key feature of the FLASH RT is the increased therapeutic index that has been confirmed with tissue‐sparing effects across various animal models, healthy organs, and several tumor types. The FLASH biological effect was first observed in electron^1^, ^3^, ^4^ and then in proton beams,^5^, ^6^, ^7^, ^8^, ^9^ showing a differential response between healthy and tumor tissue in‐vivo trials.^3^, ^4^, ^10^, ^11^ As an added benefit, by reducing the treatment time (< 500 ms), uncertainties associated to organ motion, for example, in lung, could be reduced.

The first patient treated of cutaneous lymphoma with electron FLASH RT at Lausanne Univ. Hospital^12^ was followed by the LANCE trial for skin cancers^13^ also with an electron accelerator. Meanwhile, the Cincinnati Children's Hospital Medical Center (CCHMC) performed the first proton FLASH non‐randomized trial confirmed the clinical feasibility of delivering UHDR with minimal severity of adverse effects (FAST‐01).^14^ A continuation of this trial with thoracic bone metastases is ongoing (FAST‐02).^15^. There is an effort in the medical and research community to carry out more studies to guide clinical trial designs.^16^, ^17^ In this context, there are also technological challenges associated with each radiation modality, to ensure a safe and methodical transition to clinical trials. In fact, some disagreements observed in the FLASH effects are likely influenced by differences in irradiation settings or uncertainties in achieving precise dosimetry under UHDR.^18^ In particular, to replicate the FLASH biological effect between institutions or compare radiobiological experiments, consistent and accurate dosimeter as well as dosimetry protocols are required. Moreover, spatial information of the dose distribution is often required as it allows for the verification of the radiation field characteristics and alignment, which is crucial for quality assurance. However, conventional active dosimeters show limited performance in UHDR and also restricted spatiotemporal resolutions.^10^, ^19^, ^20^

Currently, FLASH RT dosimetry is mainly performed with passive dosimeters since they are dose‐rate independent, for example, alanines, thermoluminescent dosimeters (TLDs), optically stimulated luminescent dosimeters (OSLDs), and radiochromic films,^21^, ^22^, ^23^ the latter allowing for spatial information. However, they require long post‐analysis times for dose determination and lack temporal resolution. Real‐time dosimeters and alternatives with sub‐microsecond time resolution to cope with UHDR deliveries rest with active detectors. Ionisation chambers (ICs),^24^ which adhere with the international protocols for reference dosimetry, have been explored for such beam conditions, however they saturate at UHDR^25^ due to the high charge densities generated in their cavities together with the slow drift time in the medium. Still, to adapt ICs for UHDR beams, there has been some development on either ultra‐thin plate chambers^26^, ^27^ or exploring different gas mixtures.^28^ Other alternatives being investigated are based in plastic scintillators^29^ and calorimeters.^30^ Solid‐state dosimeters based on semiconductors are good candidates as active dosimeters for FLASH RT. For instance, silicon has been studied in UHDR,^31^, ^32^ although it has lower radiation hardness than other materials. Indeed, even though its application into an array dose monitor for FLASH has been proposed,^33^ the system suffered from sensitivity loss due to radiation of 0.3%/kGy during the experiments. Diamond has been successfully utilized for point dosimetry in these conditions.^34^, ^35^, ^36^ However, detector grade diamonds are commercially available in few‐${\mathrm{mm}}^{2}$ plates, and are quoted for orders of magnitude more per area with respect to other semiconductors, making the scalability of diamond devices a challenge, constraining the use‐case of pixelated diamond devices to microdosimetry dose monitoring.^37^ In this context, silicon carbide (SiC) is a wide band‐gap (3.27 eV) semiconductor which ‐ like diamond ‐ is more radiation resistant than silicon, insensitive to visible light and has low leakage current.^38^ Unlike diamond, high resistivity silicon and SiC which are available in 4‐8 inch wafers are more affordable. SiC also shows a lower sensitivity (425 pC/($\mathrm{mGy}\cdot {\mathrm{mm}}^{3}$) for the 4H‐SiC polytype) than silicon (644 pC/($\mathrm{mGy}\cdot {\mathrm{mm}}^{3}$)), which reduces the possibility of saturation in high dose rate environments. Finally, in terms of time resolution, impacted by the saturation velocity of the electrons, SiC (2.0 $\times 10^{7}$ cm/s) is comparable to diamond (2.2 $\times 10^{7}$ cm/s) and faster than silicon (0.8$\times 10^{7}$ cm/s).^38^

The Institute of Microelectronics of Barcelona (IMB‐CNM) has developed a robust SiC technology for radiation detection. Collaborating with the ALBA and ESRF synchrotrons, the IMB‐CNM developed SiC photodiodes^39^, ^40^ that exhibit superior performance and radiation hardness compared to their silicon counterparts, even at high temperatures and under high‐fluence ion exposure.^41^, ^42^ Building on the success of reliable SiC radiation detectors, SiC diodes for UHDR featuring a 3 μm epitaxial layer and 1 mm diameter (radiation‐sensitive volume) were designed and produced at IMB‐CNM for the EU EMPIR‐UHDPulse project.^20^, ^43^, ^44^ This project aimed to develop metrological tools to establish traceability in absorbed doses in UHDR. The SiC devices were tested with pulsed electron beams of 20 MeV, with dose per pulses of 0.1 to 11 Gy with 0.5 to 3 μs pulses, the highest reported at the time with SiC, keeping a linearity better than 3% and with a low long‐term response degradation of 0.02%/kGy.^44^

Another challenge in the UHDR dosimetry is the development of suitable readout electronics. There are two main issues: the electronic saturation in current electrometers and the lack of time resolution of the pulse measurements. For the former, we have manufactured a multi‐capacitor system (12 channels) to be connected at the input of a commercial electrometer to sufficiently lower the voltage on the signal cable to keep the electrometer operational amplifier within its linear range. For the latter, the time‐resolved measurements, we have manufactured an amplifier board allowing the probing of the time structure of the pulse by means of a standard oscilloscope.

This work presents the response of a small prototype for a larger scale dose monitor (i.e., a small dose monitor) for the first time, consisting of a 4 × 4 array of SiC diodes (1 mm diameter, 2.2 mm pitch), to UHDR irradiation using 7 MeV electron pulsed beams with pulse widths ranging from 0.5 to 5 μs in the Institut Curie (France). To do so, first, the linearity of the devices was evaluated by means of a single SiC diode with doses up to 10 Gy per pulse. Secondly, the intra‐pulse time structure was characterized with the same device by varying the pulse length from 0.5 to 5 μs. Finally, the dose distribution over a total area of 70 × 50 ${\mathrm{mm}}^{2}$, with a granularity of 1 × 2.2 ${\mathrm{mm}}^{2}$, was measured using the 4 × 4 pixel technology demonstrator with pulses of 3 μs delivering 7 Gy per pulse at the central position. Both the single and 4 × 4 SiC diode configurations operated without external voltage bias. The results presented serve as a proof of concept for the ongoing development of a multi‐channel system for real‐time dose monitoring in UHDR conditions and with high spatiotemporal resolution.

## 2 MATERIALS AND METHODS

### 2.1 Sample preparation

**FIGURE 1:**
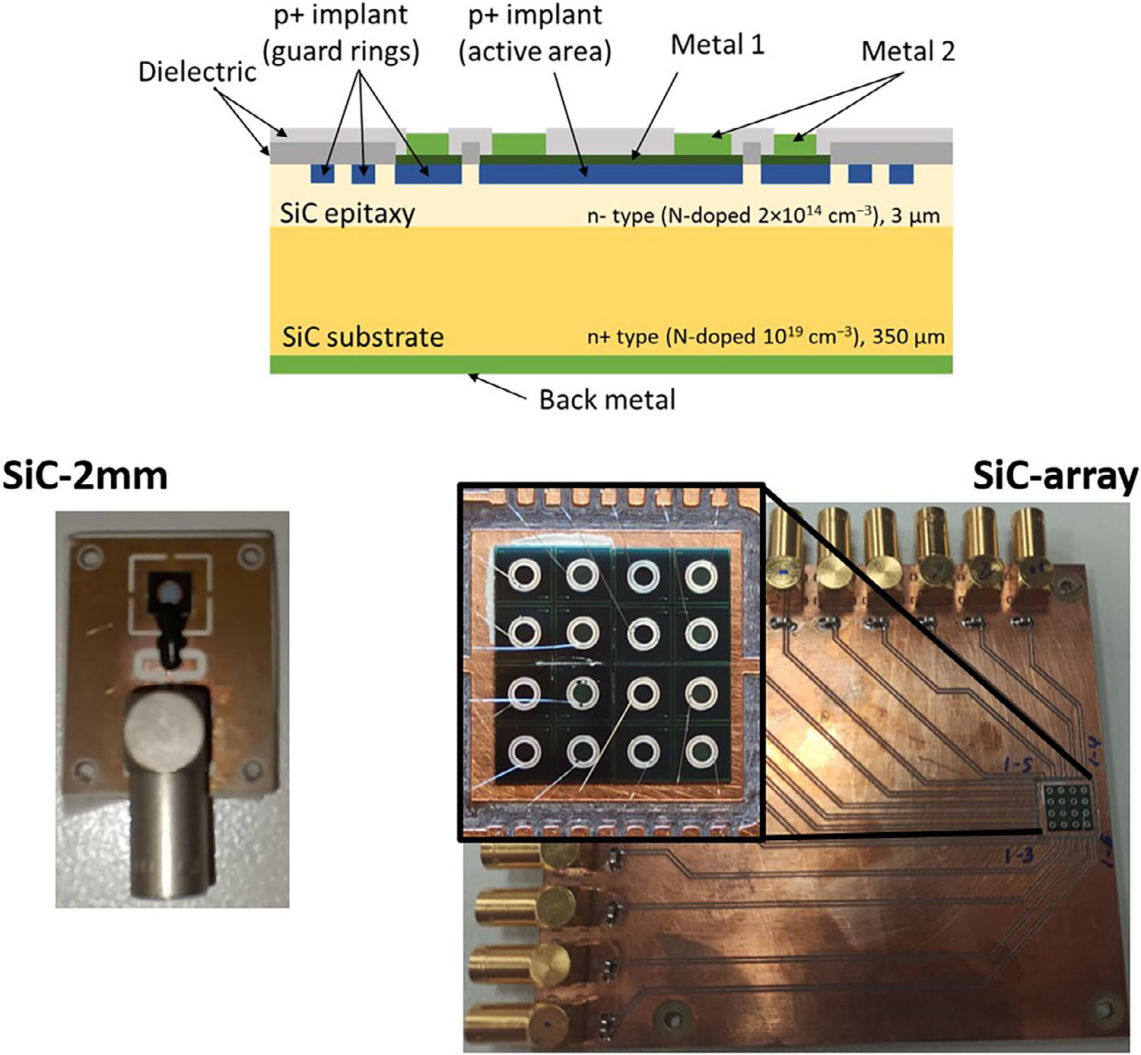
Top: Schematic of the SiC diode cross‐section. Bottom, left: 2.2 mm diameter Si diode on testing in‐house board. Bottom, right: 4 × 4 array of SiC diodes (1 mm diameter, 2.2 mm pitch) on an in‐house board.

The design, fabrication, and preliminary characterization of all the devices were done at IMB‐CNM. These were fabricated from 4‐inch 4H‐SiC wafers with a 3 μm thick high resistivity epitaxial layer over a 350 μm thick low resistivity substrate (see Figure 1, (top)). More details on the fabrication can be found elsewhere.^44^

Among the structures produced on the same wafer were a 2.2 mm diameter diode (“SiC‐2mm”) and a 4 × 4 diode array (“SiC‐array”) (see Figure 1, bottom). Each diode on the array has 1 mm diameter, with a pitch of 2.2 mm in either direction. While the active area of SiC‐2mm is completely metallized, half of the channels of the SiC‐array device were also fully metallized, while the remaining pixels were only metallized in a concentric ring on the outer region of the 1 mm diode. This variation was introduced as part of a fabrication optimization study where the metals are reduced to allow for other characterization techniques. This difference in the metallizations is not expected to have any significant effect in the response to the radiation beams used in this experiment.

The samples were glued on printed circuit boards (PCBs) with silver paste for electrical interconnection with the back side, while the front side connection was performed with wire bonds. The diodes on the array were wired to separate channels with 100 nF capacitors on a 16‐channel output PCB to allow spatial resolution measurements (see Figure 1, bottom).

Throughout the experiments, a calibrated PTW flashDiamond (PTW‐fD) dosimeter^34^, ^45^ (D8545 model) was utilized as reference detector.

Finally, with the aim of measuring the beam profile independently, ${\mathrm{GAFChromic}}^{TM}$ EBT‐XD films^46^ were used. The optimal dose range of these films is between 0.4 Gy and 40 Gy, with dose uniformities of less than ±3% in dose, and were handled according to the recommendations of AAPM 68 TG‐235.^47^

A summary of the dosimeter devices utilized in the experiments can be found in Table 1.

**TABLE 1:** List of devices utilized in the experiments.

Sample	Geometry	Characteristics	Readout
SiC‐2mm^44^	Single diode	1‐channel	PTW unidos
	2.2 mm diameter		e‐meter
SiC‐array^44^	4×4 diode array	16‐channel array	PTW multidos
	1 mm diameter	demonstrator	e‐meter
PTW‐fD^34^, ^45^	Flash	Reference	PTW unidos
	diamond		e‐meter
EBT‐XD^46^	Gafchromic	Reference	Flatbed RGB
	film		scanner

### 2.2 Experimental set‐up

Irradiations were performed at the pre‐clinical ElectronFlash electron linear accelerator (SIT technologies) at the Institute Curie.^48^ This accelerator is able to deliver electrons of 5 or 7 MeV with varying pulse widths of 0.5 to 5 μs at rates of up to 250 Hz. At the LINAC aperture, an exchangeable cylindrical applicator (AP) is installed in order to focus the electron beam. The applicators utilized in this experiment ranged from 120 to 40 mm diameter and a distance ranging from 1100 to 600 mm respectively from the source, allowing to vary the dose per pulse delivered by the machine from ∼0.5 to over 10 Gy per pulse respectively. A beam current transformer (BCT), from Bergoz Instrumentation, located just upstream of the accelerator output window, allows to monitor its throughput at per pulse basis.

**FIGURE 2:**
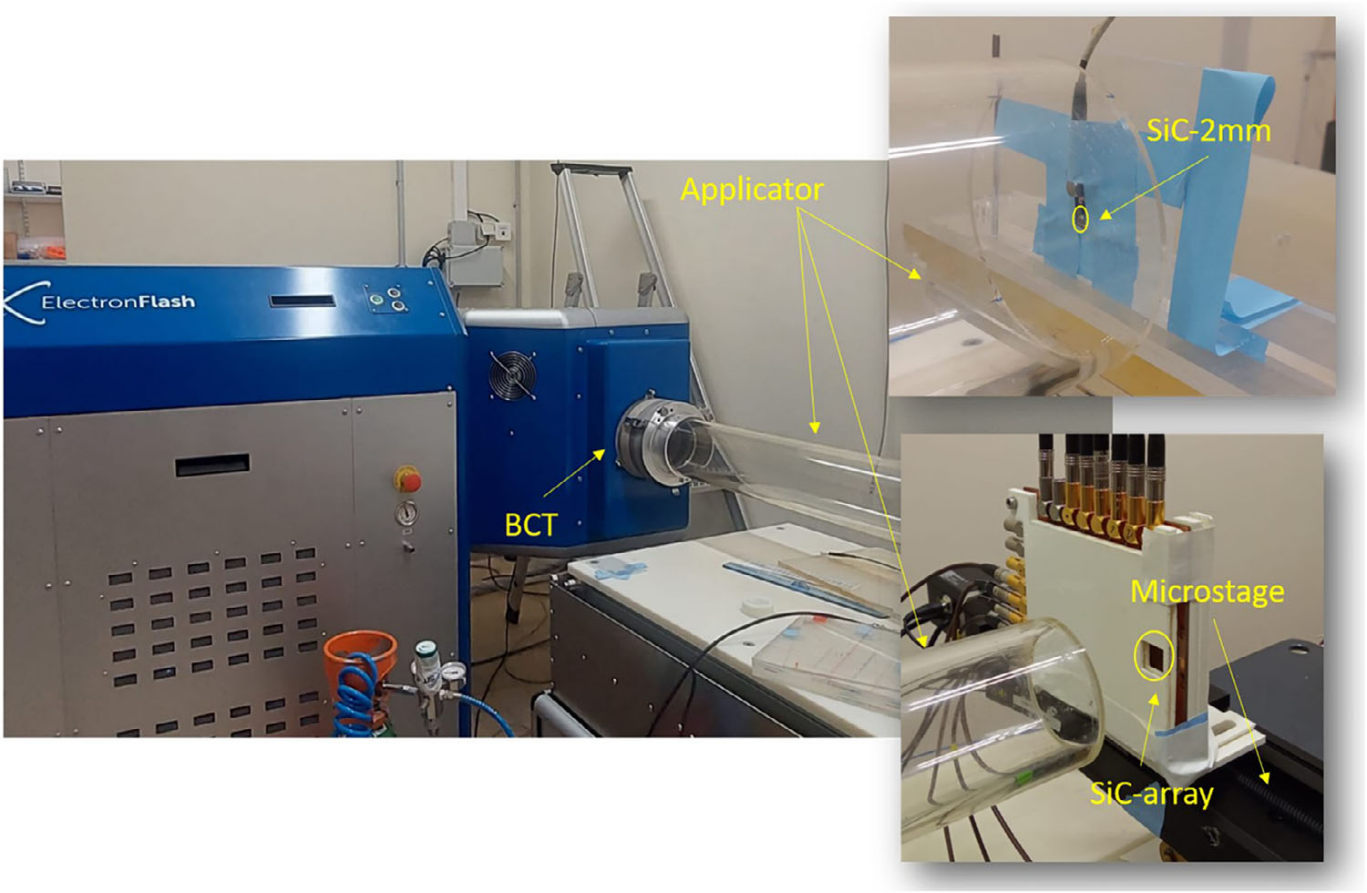
Picture of the ElectronFlash LINAC (left) and the individual SiC (2 mm diameter) and the 2 × 2 array (1 mm diameter) placed on a movable micro‐stage to cover larger surfaces utilized for this work (right).

The devices were located at the output of the applicator (see Figure 2), and replaced with the PTW‐fD sample when reference dose measurements were required. The detectors were read out with PTW Unidos and Multidos electrometers for the single diodes and the 4 × 4 array respectively. A 100 nF capacitor was connected in parallel at each channel input, either by means of a customized aluminum‐shielded 12‐channel adapter for the PTW Multidos electrometer or a single‐channel adapter for the PTW Unidos, which is offered by PTW as part of the flashDiamond package for UHDR. The capacitors were added to suppress spikes, preventing voltage‐induced interference with the diode. The charge integrated by the electrometer was digitized and acquired with a custom python code, recording the total charge collected by the single‐channel or SiC array for each pulse.

The SiC‐2mm device was fixed onto a PMMA slab to measure the linearity of its response and its time‐resolved readout (Figure 2, top‐right), and the SiC‐array device was mounted on a movable microstage to measure the dose distribution and profile (Figure 2, bottom‐right). With it, the charge was measured in 1 mm steps across the beam. The EBT‐XD radiochromic films were irradiated at the same distance as the active area of the SiC‐array detector. The films were scanned with a RGB flatbed scanner (Epson Perfection V850 Pro) with a resolution 96 dpi. The tagged image file (tiff) was analyzed to determine the relative dose after applying the calibration in the red channel.

## 3 RESULTS

The SiC diode technology was first characterized with the single‐channel SiC‐2mm detector for simplicity by measuring the its response as a function of dose per pulse (DPP). Time‐resolved measurements were carried out with the same diode. Finally, the 4 × 4 diode array (1 mm diameter, 2.2 mm pitch) mounted on a movable microstage was utilized as a technology demonstrator for a dose monitoring system under development.

### 3.1 SiC diode linearity

**FIGURE 3:**
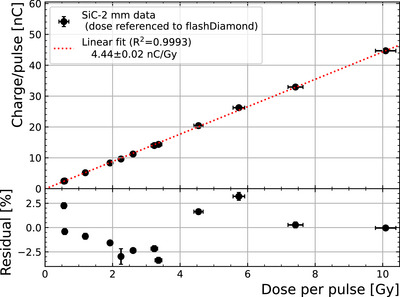
Charge per pulse recorded from the device SiC‐2mm as a function of the dose per pulse obtained from the PTW‐fD reference dosimeter up to 10 Gy per pulse. The residuals from the fit are shown above.

Figure 3 shows the results of the linearity measurements compared to the PTW‐fD reference dosimeter. The uncertainty of the SiC response was calculated statistically after at least three measurements per condition. This yields uncertainties lower than 1%. On the other hand, the absolute reference dosimetry uncertainty from the PTW‐fD is taken as 3% (*k* = 1). A linear fit was applied to the data up to a DPP of 10 Gy, which is the upper limit to which the PTW‐fD dosimeter used for reference is known to provide accurate readings.^34^, ^35^ The residuals indicate a linearity better than 3.5% at least up to 10 Gy per pulse, which is comparable to the PTW‐fD specifications, even though the packaging and sensor geometry were not optimized for low noise measurements. The SiC‐2mm diode response obtained from the fit in this DPP range is (4.44 ± 0.02) nC/Gy.

After exploring the range in DPP up to 10 Gy, measurements were repeated in the low range of DPP (included in Figure 3), with no significant deviation observed. Thus, no radiation damage was observed throughout the experiment, with an accumulated dose exceeding 1 kGy. This result is compatible with the reported 0.02%/kGy sensitivity loss in similar experiments.^44^

### 3.2 Time‐resolved measurements

An in‐house current‐to‐voltage converter with an amplification factor of 100 V/A was connected to the output of the SiC‐2mm device. The amplified signal was then fed to a Teledyne Wavesurfer 4054HD oscilloscope operating at 5 GSa/s. On the same oscilloscope, the output of the accelerator's BCT was also monitored.

**FIGURE 4:**
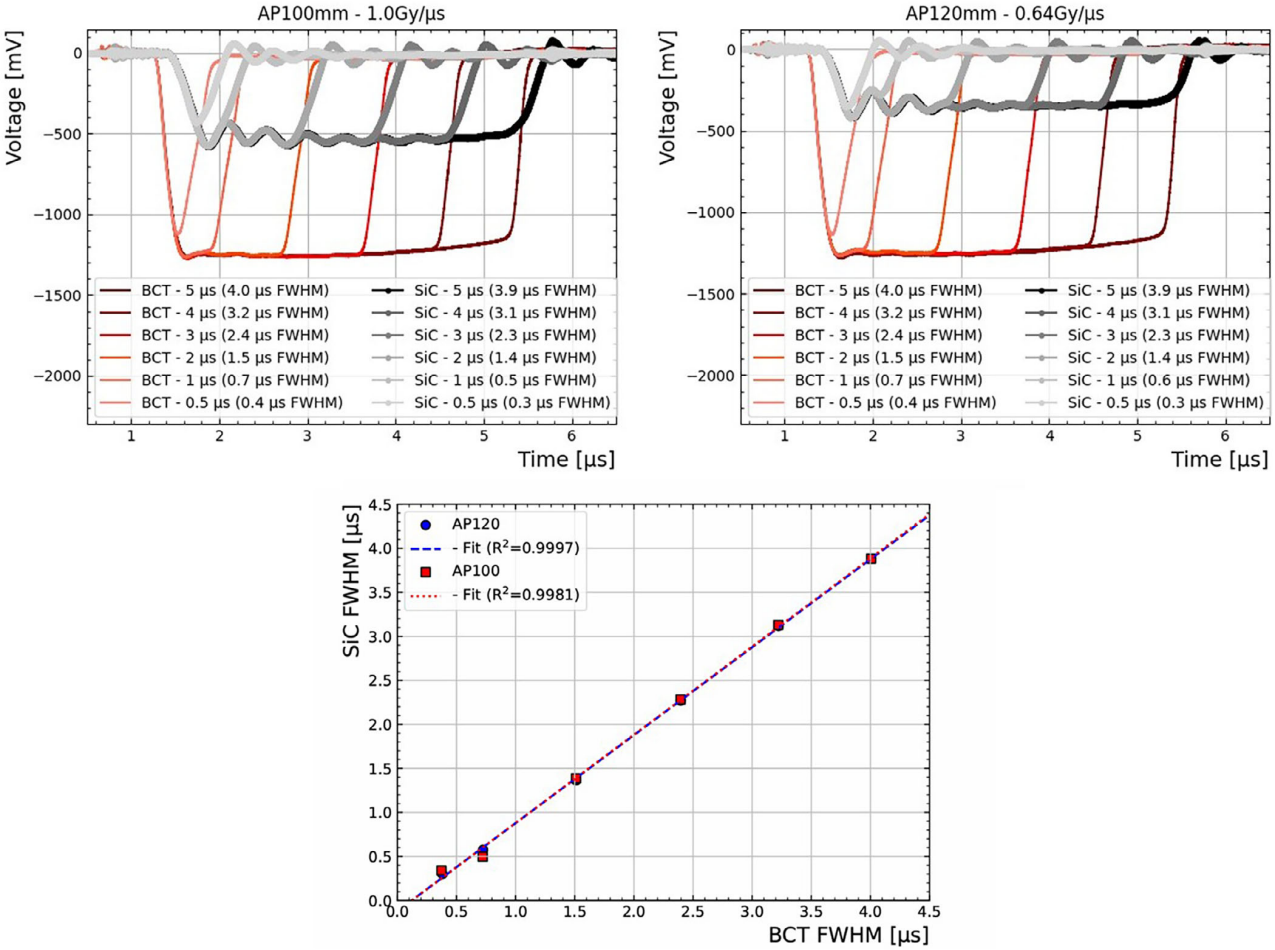
Pulses recorded from the SiC‐2mm device and the beam current transformer (top) with an applicator of 120 mm (left) and 100 mm (right). Above, a correlation between the FWHM for each condition is shown.

The device under test was irradiated with 3 pulses and varying pulse lengths from nominally 0.5 to 5 μs. Figure 4(top) shows the resulting pulses from the prototype dosimeter and the BCT, with the applicator of 120 mm (Figure 4(top, left)) equivalent to 0.64 Gy/μs and 100 mm (Figure 4(top, right)) delivering a dose rate of 1.0 Gy/μs. In all cases, the pulse lengths are consistent for each event. The pulse amplitude from the SiC‐2mm sample is proportional to the dose rate, as expected. A delay of about 250 ns is observed between the SiC signal and the BCT signal, possibly due to a combination of the current to voltage amplifier electronics, difference in cable lengths and the electron travel distance. Indeed the BCT measures the electron current at the exit window of the accelerator, whereas the SiC diode measures the signal at the applicator exit point.

Moreover, as can be seen in Figure 4(bottom), the FWHM of the pulses in both systems match, demonstrating that the SiC pulse measurements are consistent with the BCT reference. However, the device signal shows oscillations, likely due to an impedance mismatch in the in‐house amplifier board, which will be addressed in future iterations.

### 3.3 Technology demonstrator of small dose monitor

**FIGURE 5:**
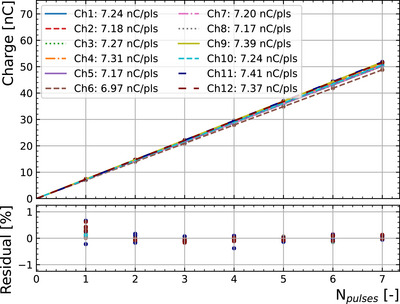
Charge measured for the 12 channels as a function of number of pulses of 3 μs using the 40 mm applicator. The responses are indicated for each channel which are used to correct for the difference in sensitivities (charge per pulse).

The SiC‐array (see Figure 2, bottom‐right) was mounted on a micrometer‐precision movable stage perpendicular to the beam direction, positioned approximately at 47 mm from the 40 mm applicator exit and with its active area aligned to the beam center. Twelve pixels were connected to the PTW Multidos electrometer for readout. In order to quantify possible differences in responsivity between the pixels, the linearity of each pixel was measured as a function of the number of pulses (with 3 μs width) with the device centered in the central axis. Figure 5 shows the integrated charge for each of those pixels as a function of the number of pulses, along with their corresponding linear fits. The slope ($R_{i}$) of each pixel (i) fit was used to correct the measured charge ($Q_{\text{meas}}$) for the sensitivity difference relative to the average response $\bar{R}$ according to the formula: $Q_{\text{corr},i}=Q_{\text{meas},i}\bar{R}/R_{i}$. The dose across the coverage of the detector is assumed to be uniform (±4.4 mm across either direction). The maximum deviation between individual diode responses and the average is of 4%, or 2% excluding the outlier pixel (i.e., Ch 6).

**FIGURE 6:**
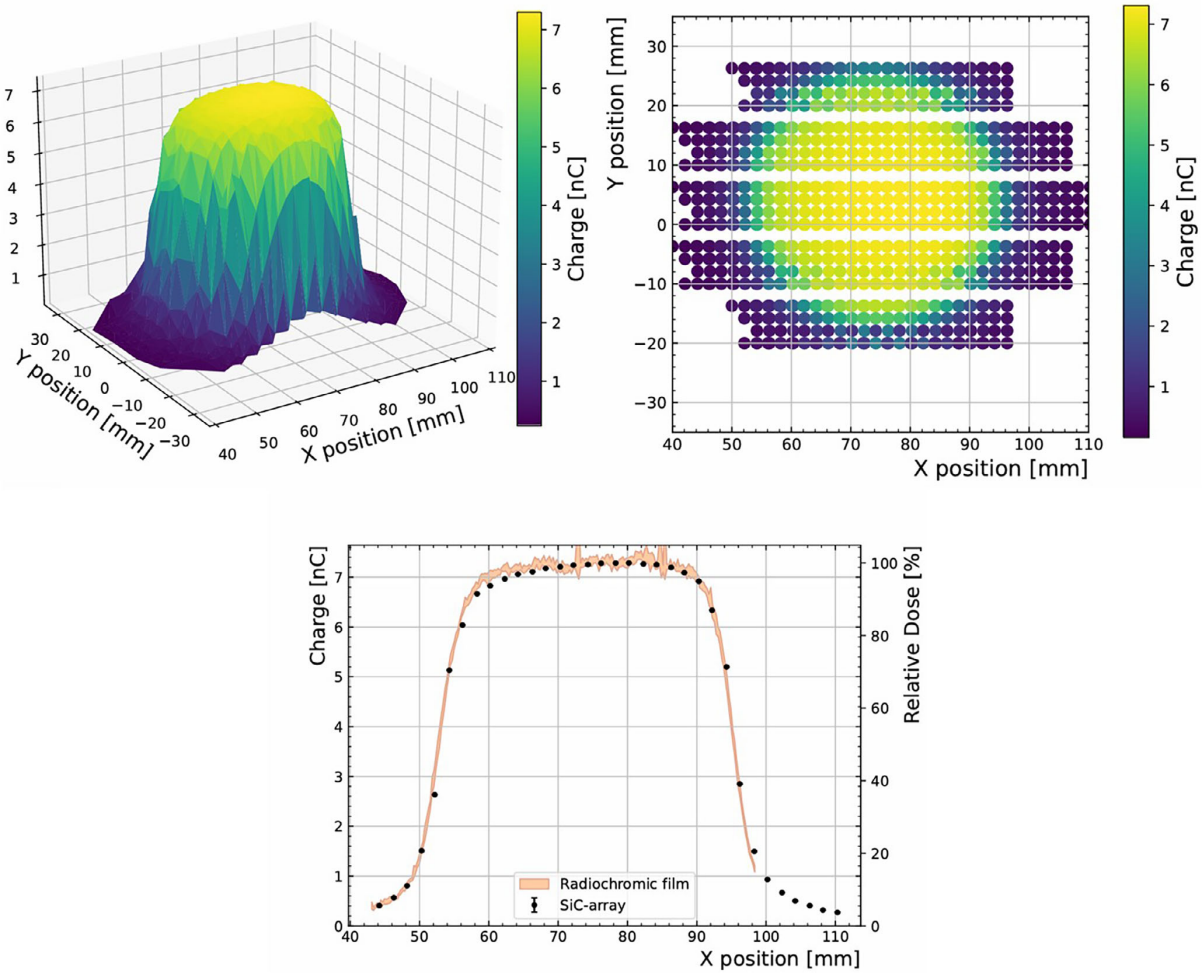
Top: Charge measured with the SiC‐array as a function of position obtained with the SiC‐array, using a 40 mm applicator. The horizontal gaps in the map (top, right) are a consequence of the 1 cm vertical steps being larger than the total detector active area. Bottom: Slice at y=2 mm of the charge measured with the SiC‐array with the relative dose as measured with a radiochromic film overlayed (orange). All charges are corrected for difference in sensitivities between pixels.

The dose distribution at UHDR was measured using the SiC‐array by recording the integrated charge from each pixel with pulses of 3 μs along the horizontal and vertical direction, perpendicular to the beam direction, with a dose of 7 Gy per pulse at the central position. Horizontally, a total distance of up to 60 mm was covered in 4 mm steps by means of the micrometer stage. Vertically, the position was adjusted manually over 40 mm in 10 mm steps to maximize coverage of the device area. A radiochromic film was later irradiated in the same conditions and distance for reference. Figure 6 (top) shows the dose distribution map (in terms of SiC‐collected charge) obtained with the 4 × 4 SiC array. Figure 6(bottom) shows the measured beam profile across the equator of the applicator. The relative dose profile measured with an EBT‐XD radiochromic film, averaged around ±1.1 mm around the center of the beam to account for potential misalignment coinciding with the pixel pitch, shows a good agreement with the SiC profiles, as quantified with collected charge or relative dose, respectively.

**FIGURE 7:**
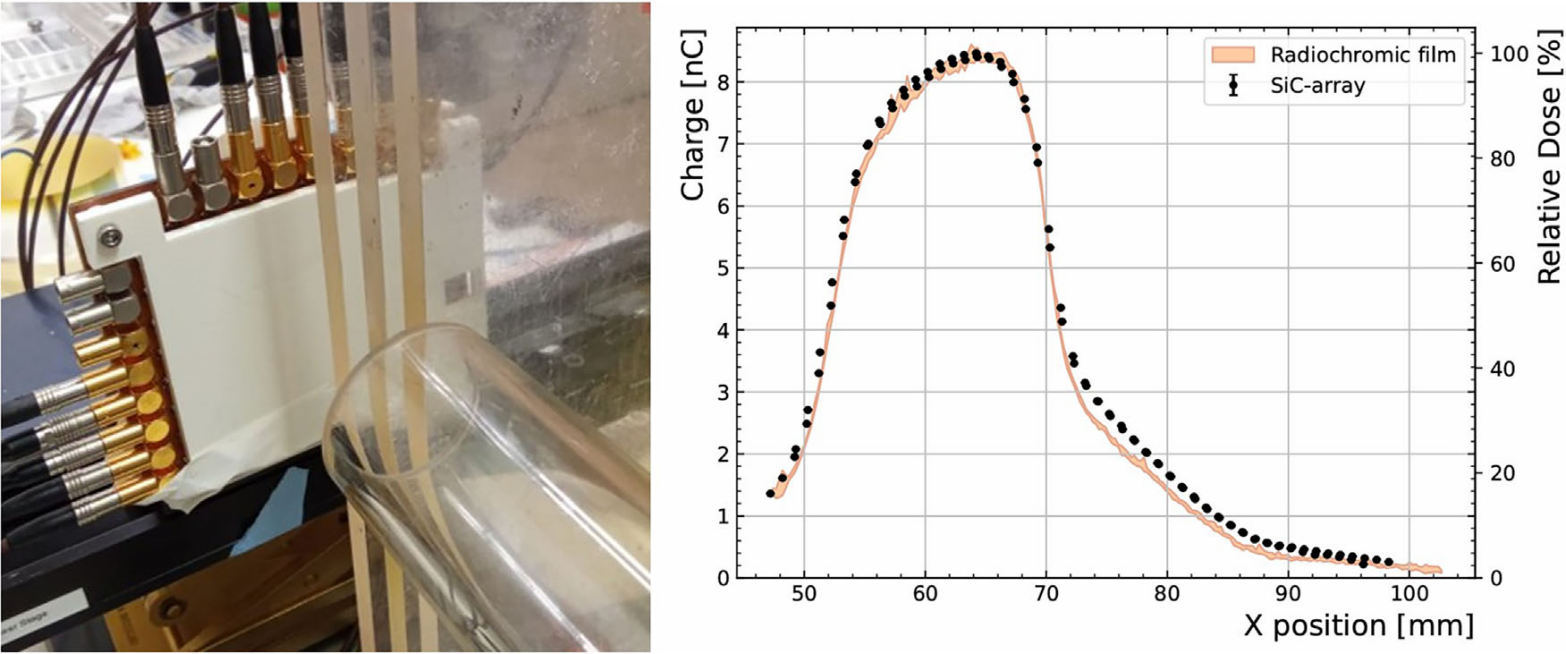
Left: Picture of set‐up including stepped layers of PMMA between the dose monitor prototype and the 40 mm applicator. Right: Charge corrected for difference in sensitivities of each pixel as a function of position obtained with the SiC‐array. The relative dose as measured by a radiochromic film at the same distance from the applicator is overlayed (orange).

Similarly, with the goal to qualify the sensitivity to dose conditions simulating animal irradiation with bolus, a series of three 1 cm thick PMMA slabs was placed downstream of the applicator exit (for reference, the PDD utilizing this facility was characterized elsewhere^48^). The slabs were positioned so that the beam is covered by 0, 1, 2 and 3 cm layers of PMMA (see Figure 7(left)), resulting in a heterogeneous beam profile. The response of the detector to the 3 μs pulses across a 50 mm range, covered in 1 mm steps, is shown in Figure 7(right), alongside the dose profile obtained by means of a radiochromic film. From these results, it is possible to observe the different doses resulting from the different thicknesses of PMMA.

The obtained distribution is consistent to that measured with radiochromic films, demonstrating the capability of the system to accurately reproduce the beam profile under UHDR conditions. Minor discrepancies are observed, which could be attributed to a slight misalignment or tilt of the radiochromic film with respect to the original position of the detector active area.

## 4 DISCUSSION

The SiC semiconductor is an excellent candidate for UHDR dosimetry due to its extreme radiation hardness, fast charge collection, and excellent noise performance. Based on these intrinsic features, a technology demonstrator of a small dose monitor array has been produced, utilizing the SiC diodes that have already shown a good performance for 20 MeV electron beams from 0.5 to 3 μs at 5 Hz in the metrology PTB facility.^44^

In this work, a dose monitor prototype based on a 4 × 4 SiC diode matrix was shown for first time. Albeit the limitation on readout electronics and channel availability, up to 12 pixels were simultaneously measured. These results constitute the first dose 2D map with SiC sensor in electron FLASH beam conditions (Figure 6). There is only one work from Schönfeld et al.,^33^ showing a 2D detector array based on silicon diodes. With an optimized electrometer system for the high dose per pulse and pulse lengths, the pixels show a good accuracy. Moreover, the pixels show consistent response to non‐uniform irradiation fields (Figure 7). A system containing more channels is under development with its corresponding readout electronics.

The linearity study yields a response of 4.44±0.02 nC/Gy. Similar detectors with same epitaxy and 1 mm diameter yield a response of 1‐1.2 nC/Gy^44^ which is consistent with the results from this study when scaling for the active area of the 2.2 mm diameter single SiC diode. This flexibility in design would allow, for instance, a system with a large detector area for measurements in the periphery (or out‐of‐the‐field), since it will be also crucial for the patient radiation protection, together with smaller ones to measure UHDR dose distributions in‐the‐field.

A good linearity better than 3.5% up to 10 Gy per pulse is achievable at 0 V, measured with a dosimeter fabricated with the same technology as the array prototype, comparable with an absolute dosimeter (PTW‐fD), and no indication of saturation is observed even though the signals generated within the SiC active volume (425 pC/$\mathrm{mGy}\cdot {\mathrm{mm}}^{3}$) are double to what a similarly sized diamond detector would observe (259 pC/$\mathrm{mGy}\cdot {\mathrm{mm}}^{3}$). Although this work exposes the SiC dosimeters to a maximum of 2.5 MGy/s instantaneous dose rate (10 Gy per pulse), other experiments with low‐energy proton UHDR have explored higher doses per pulse (up to 25 Gy per pulse)^49^ and instantaneous dose rates (4.6 MGy/s)^50^ with no indications of saturation. In other controlled electron facilities,^44^ these detectors have withstood a dose rate of 3.8 MGy/s (11 Gy per pulse) with no signs of saturation. The limitation of the technology is still being investigated.

Current diamond single‐point dosimeters for UHDR^34^, ^45^ have linear response of up to at least 10 Gy per pulse on electron beams, but diamond remains costly to manufacture, suffers from production repeatability challenges, and is unavailable in large wafer sizes. These factors hinder its scalability for widespread evaluation of dose maps covering several centimeters of area. Silicon detectors have also been proposed for UHDR dosimetry elsewhere^32^ and tested up to 10 Gy per pulse with electrons, however, that work reports a requirement of high bias voltage to be applied in order to maintain linearity. Moreover, silicon has lower radiation tolerance than SiC. A sensitivity loss from 20 MeV electrons of 0.02%/kGy^44^ has been observed with SiC, while commercial silicon dosimeters report 0.1%/kGy with 6 MeV electrons.^51^ Indeed, no significant change in sensitivity was observed during the experiment after accumulating a dose of more than 1 kGy. This reduces the amount of calibrations required for the final system over its lifetime. Other designs of single‐point SiC devices have been explored for UHDR dosimetry.^52^, ^53^ However, owed to a shallow epitaxy of 3 μm of the dosimeters in this work, the built‐in voltage from the PN‐junction is enough to deplete the full volume, which allows to reliably achieve this performance with 0 V applied bias voltage with a well‐defined active volume. By removing the high voltage requirement to operate the detector, the design and development of compatible electronics is simplified.

The verification of the pulse time structure is of relevance as studies have shown that the biological effect could depend on the temporal structure of the treatment.^9^ This work demonstrates the ability of the detector to resolve sub‐microsecond FLASH pulses (below microseconds), useful for the verification of the radiation pulse structure at the measurement point in real‐time. The capability of SiC‐based detectors to achieve sub‐microsecond resolution is to be expected thanks to the high mobility of the charge carriers in the material. Notice that the BCT is installed at the output of the accelerator, and thus it is not able to account for differences in applicators and distance. In contrast, the system proposed here allows the possibility for instantaneous pulse discrimination at UHDR and dose rate measurements at the same time. This has been also achieved in silicon^32^, ^33^ by the use of the TERA08 ASIC^54^ at 100 V, and with the EDGE detector at 0 V, the later reporting non‐linearities at high dose rates.^33^ Similar attempts have been achieved with diamond detectors,^36^ in which the pulse structure measured by the diamond matches that of the reference BCT detector using a commercial trans‐impedance amplifier. Work is ongoing to allow for multiple‐channel pulse measurement, since in most cases, the pulse measurement requires an oscilloscope^32^, ^36^, ^55^ which limits the granularity by the number of channels. This aspect is of vital importance to validate the dose rates delivered in clinical trials with sub‐millimeter spatiotemporal resolution. For example, recently a CMOS camera was used for retrospective QA validation in the FAST‐01 clinical trial,^56^ showing the importance of this kind of tools for further QA.

Other solutions for dose monitors have been reported. For instance, the FLASH Profiler,^33^ consisting of 139 silicon diodes set at a higher pitch of 4 mm. This device was optimized for UHDR up to 5.5 Gy per pulse, although a sensitivity loss of 0.3%/kGy was reported with 20 MeV electrons, larger than the 0.018%/kGy reported for SiC elsewhere.^44^ Also with silicon, a prototype of a silicon‐based monolithic array was proposed and produced by Filipev et al.^57^ covering an area of 58 × 58 ${\mathrm{mm}}^{2}$, tested in a conventional RT LINAC, but not tested for UHDR. The proposed system based on SiC aims to cover the same needs for FLASH by measuring the 2D dose distribution with a radiation resistant instrumentation and higher granularity. Finally, diamond arrays have been produced for microdosimetry. Loto et al^37^ reported a system able to measure the LET and dose rate simultaneously for proton beams. Even though such a system is well suited for microbeam characterization, scaling the production to cover wider beams is challenging due to the cost of the material.

## 5 CONCLUSION

The SiC‐2mm single diode working at 0 V showed a response linearity better than 3.5% up to 10 Gy per pulse in a pre‐clinical electron accelerator facility. It also showed a good temporal resolution, able to discriminate pulses from all the pulse width range offered by the electron LINAC (0.5 to 5 μs) using an in‐house current to voltage transformer and an oscilloscope. The pulse widths measured matched those from the reference BCT of the accelerator. The results demonstrated that the SiC technology developed at IMB‐CNM can assess instantaneous dose rates as well as the temporal structure of the pulses. It is a cost‐effective alternative to silicon and diamond for UHDR dosimetry due its fast response and radiation hardness.

The technology demonstrator, consisting of a SiC 4 × 4 array (1 mm diameter, 2.2 mm pitch), connected to a multi‐channel readout system and mounted on a movable microstage, showed its potential as a dose monitor for FLASH RT. A dose map of a 70 × 50 ${\mathrm{mm}}^{2}$ area was quantified under UHDR conditions (7 Gy per pulse, 3 μs pulse width) with 7 MeV electrons. To the best of our knowledge, this is the first time that a 2D dose distribution is measured in real‐time under UHDR irradiation. In addition, dose profiles were measured and cross‐checked with those obtained with radiochromic films at similar positions, demonstrating also the array capacity as accurate profiler. The device was also verified with an inhomogeneous beam, simulating an animal irradiation bolus. The results showed the viability of scaling up to a larger‐area prototype for further 2D dose monitor. In this line, smaller SiC diodes with 600 μm diameter have been manufactured to produce a new, larger scale, dose monitor, which will improve the spatial resolution and decrease the noise by reducing the capacitance. The readout electronics for the new demonstrator ‐ which consists of 121 SiC diodes spanning 24 mm ‐ has been developed to allow for multi‐channel measurements by means of commercially available ICs for signal amplification, which uses an FPGA for the read‐out of the full array. Time‐resolved capabilities are to be integrated. Characterization of the system in UHDR beams is currently ongoing.

## CONFLICT OF INTEREST STATEMENT

The authors declare no conflicts of interest.
